# Differential Regulation of Antagonistic Pleiotropy in Synthetic and Natural Populations Suggests Its Role in Adaptation

**DOI:** 10.1534/g3.115.017020

**Published:** 2015-02-23

**Authors:** Anupama Yadav, Aparna Radhakrishnan, Gyan Bhanot, Himanshu Sinha

**Affiliations:** * Department of Biological Sciences, Tata Institute of Fundamental Research, Mumbai, 400005, India; † Department of Molecular Biology and Biochemistry, Rutgers University, Piscataway, New Jersey 08854

**Keywords:** antagonistic pleiotropy, QTL, gene−environment interaction, epistasis, Ras/PKA pathway

## Abstract

Antagonistic pleiotropy (AP), the ability of a gene to show opposing effects in different phenotypes, has been identified in various life history traits and complex disorders, indicating its fundamental role in balancing fitness over the course of evolution. It is intuitive that natural selection might maintain AP to allow organisms phenotypic flexibility in different environments. However, despite several attempts, little evidence exists for its role in adaptation. We performed a meta-analysis in yeast to identify the genetic basis of AP in bi-parental segregants, natural isolates, and a laboratory strain genome-wide deletion collection, by comparing growth in favorable and stress conditions. We found that whereas AP was abundant in the synthetic populations, it was absent in the natural isolates. This finding indicated resolution of trade-offs, *i.e.*, mitigation of trade-offs over evolutionary history, probably through accumulation of compensatory mutations. In the deletion collection, organizational genes showed AP, suggesting ancient resolutions of trade-offs in the basic cellular pathways. We find abundant AP in the segregants, greater than estimated in the deletion collection or observed in previous studies, with *IRA2*, a negative regulator of the Ras/PKA signaling pathway, showing trade-offs across diverse environments. Additionally, *IRA2* and several other Ras/PKA pathway genes showed balancing selection in isolates of *S. cerevisiae* and *S. paradoxus*, indicating that multiple alleles maintain AP in this pathway in natural populations. We propose that during AP resolution, retaining the ability to vary signaling pathways such as Ras/PKA, may provide organisms with phenotypic flexibility. However, with increasing organismal complexity AP resolution may become difficult. A partial resolution of AP could manifest as complex human diseases, and the inability to resolve AP may play a role in speciation. Our findings suggest that testing a universal phenomenon like AP across multiple experimental systems may elucidate mechanisms underlying its regulation and evolution.

Antagonistic pleiotropy (AP) is the ability of a gene to affect two or more phenotypes in opposite directions. First proposed with reference to reproduction and lifespan (Williams 1957), antagonism has now been identified across various traits such as cancer (Carter and Nguyen 2011; Hu *et al.* 2007), senescence (Ungewitter and Scrable 2009), immunity (Vincent and Sharp 2014), metabolic disorders, and sexual antagonism (Rice 1996; Foerster *et al.* 2007). One of the principal roles of AP is balancing active growth and cellular robustness (Holzenberger *et al.* 2003; Lehtinen *et al.* 2006; Dowling and Simmons 2009; Finch 2010; Campisi *et al.* 2011). Oxidative and DNA damage, which accumulate as a consequence of proliferation or from a defense response against pathogens (Lambeth 2007), eventually compromises regenerative ability, resulting in reduced fitness (Apel and Hirt 2004). However, although enhanced stress resistance is associated with increased robustness, it leads to compromised growth (Honda and Honda 1999; Holzenberger *et al.* 2003; Longo and Finch 2003), requiring the genome to play a balancing act, which results in AP.

The existence of such trade-offs in multiple phenotypes results in a paradigm where no single mutation can be advantageous for all phenotypes in all environments (Fisher 1930; Levins 1968) and has major implications on rates and limits of adaptation (Orr 2000, 2005). This has been one of the central components of evolutionary theories, especially those concerning life history traits (Stearns 1989). Although genes with antagonistic effects on diverse phenotypes have been identified (Longo and Finch 2003; Wang *et al.* 2005; Fernandez 2010), the contribution of these trade-offs in shaping adaptation, and thus modulating phenotypic variance is unclear (Hedrick 1999; Roff 2000; Leroi *et al.* 2005; Anderson *et al.* 2011; Wit *et al.* 2013). This is primarily due to limited evidence for the abundance of trade-offs in natural populations (Leroi *et al.* 2005). This could be either because mutations with detrimental effects are not tolerated and hence selected out of the evolving population or because they show small effect trade-offs and hence are not identifiable in mapping studies. Alternately, it is possible that alleles showing high trade-offs exist but, over the course of evolution, are compensated by other mutations, mitigating (resolving) the trade-offs by reducing the associated fitness cost and compromising their identification. This would then imply that in natural populations, variation is regulated by sets of genes, which balance conditional antagonism and general fitness (Olson-Manning *et al.* 2012).

We used budding yeast, *Saccharomyces cerevisiae*, as a system to study such trade-offs in growth, in favorable, oxidizing, and DNA-damaging environments. It is well known that different genetic loci regulate traits such as growth and sporulation for strains adapted to laboratory conditions compared with natural strains (Nogami *et al.* 2007; Fraser *et al.* 2012). In addition, different genetic variants can regulate variation in the same phenotype in different strains (Cubillos *et al.* 2011), emphasizing the importance of the evolutionary history of a strain. Hence, to understand the role of AP in adaptation and its genetic regulation, we performed a meta-analysis to investigate trade-offs in three different experimental paradigms: a genome-wide deletion collection (constructed in a long-established laboratory strain, BY), natural strains (diverse natural isolates of the SGRP collection, Liti *et al.* 2009), and quantitative trait locus (QTL) mapping in a synthetic recombinant population derived by crossing the laboratory strain (BY) and a wine isolate (RM), which represents an intermediate between the other two experimental populations (Bloom *et al.* 2013). Although both the deletion collection and the synthetic population showed trade-offs, very little AP was observed in the natural isolates. Among the synthetic datasets, a greater prevalence of environment dependent trade-offs was observed in the allelic variants than the deletion collection. These observations emphasize the importance of the role of mutations over the role of genes, in regulating AP (Paaby and Rockman 2014). Using two-QTL mapping, we demonstrated a possible resolution of this AP in parental combinations, *i.e.*, the parental combinations had less AP compared with each allele independently, suggesting that although a particular allele was capable of showing AP across environments, its effect was mitigated by other variants present in the parental background. In the deletion collection analysis, the highest trade-offs were observed in genes involved in mitochondrial and cellular organization. However, in the recombinant population, we found that *IRA2*, a negative regulator of the Ras/PKA signaling pathway, showed significant AP. Moreover, we found that several other genes of the Ras pathway showed balancing selection in both *S. cerevisiae* and *S. paradoxus*. These results suggest that in unicellular organisms, AP in key growth pathways such as the Ras/PKA pathway (Olson-Manning *et al.* 2012) may be resolved over time by spreading the fitness cost to downstream genes, allowing phenotypic flexibility in changing environments. Such a mechanism for resolving AP would allow organisms to retain the ability for rapid phenotypic switching, and may provide a rationale for the formation of regulatory hubs. Partially resolved AP (Dettman *et al.* 2007) or “frozen in” resolutions involving multiple genes may also play a key role in speciation. Our results exemplify how investigating a universal phenotype like AP across different strains and experimental systems can help provide insights into its regulation and evolution.

## Methods

### Calculating AP in deletion data

Growth data and associated microarray files for a genome-wide yeast homozygous deletion collection (Hillenmeyer *et al.* 2008) for rich (YPD) and stress [hydrogen peroxide, paraquat, hydroxyurea, and 4-nitroquinoline 1-oxide (4NQO)] conditions were downloaded from http://chemogenomics.stanford.edu/supplements/global/download.html. The normalized gene intensity values from the microarray data were used for analysis. The intensity values were scaled to zero mean and unit variance and then compared between rich growth YPD and a stress condition, as well as between the pairs of stress conditions. Genes with expression values less than −0.5 in one environment and greater than +0.5 in the other environment were shortlisted as genes showing a trade-off. Similar analysis was done using 0.75 SD as the cut-off. The degree of antagonism was calculated as the ratio of the number of genes showing trade-offs in the two environments to all genes with a significant trade-off in either environment separately. Gene ontology (GO) enrichment was done using YeastMine (Balakrishnan *et al.* 2012).

### Calculating AP in SGRP strains

Growth values obtained from Warringer *et al.* (2011) for each environment were scaled to zero mean and unit variance. Strains with values less than −0.5 or greater than +0.5 were considered significantly variable from the population mean. A strain showing growth greater than +0.5 SD in an environment and less than −0.5 SD in any other environment was considered as showing a trade-off and vice versa.

### Segregant growth data

The raw growth data analyzed in this study was derived from a study by Bloom *et al.* (2013), in which the experimental procedures are described in detail. The data we used were generated for 1008 segregants derived from a cross between *S. cerevisiae* strains BY (a laboratory strain identical to one use for genome-wide deletion collection) and RM11-1a (a wine isolate, indicated as RM). These segregants were grown in 46 different conditions. Of these, we studied the following 12 conditions: rich media with 2% glucose (YPD and YNB), other fermentable carbon-sources (lactose, raffinose, and xylose), nonfermentable carbon-source (ethanol), oxidizing agents (hydrogen peroxide, diamide, and paraquat), and DNA-damaging agents (hydroquinone, hydroxyurea, and 4NQO). See Supporting Information, File S1 for more information. Pairwise Pearson correlation was calculated for all the pairs of the selected environments and clustered using the correlation as distance (see Figure S1 and File S1).

### QTL mapping

The single environment QTL, gene−environment interactions (GEIs), and two-QTL mapping was carried out as described previously (Bhatia *et al.* 2014). Slope QTL were mapped by single QTL mapping using the slope between two environments as a phenotype. Slope was calculated using the standardized phenotype values for each pair of environments. Loci with R/qtl GEI p-value < 0.1 and slope QTL p-value < 0.05 were considered as significant GEI loci. For the targeted two-QTL interaction mapping, the markers with a p-value < 1.0 from single environment QTL mapping and the scale and crossover markers obtained from both GEI and slope QTL mapping at a p-value < 1.0 and a distance less than 60 kb were collated. See File S1 for details.

### Classification of GEI loci

To categorize significant GEI loci in three categories (environment specific, scale, and antagonistic), a *t*-test was carried out between the two alleles in each environment for each locus. The p-value was corrected for the maximum number of antagonistic interactions per environment (average of 10). Thus, a cut-off of 0.005, *i.e.*, 0.05/10, was considered significant. Environment specific QTL were loci significant in only one environment, whereas scale QTL were loci significant in both environments, with effects in the same direction. Finally, antagonistic QTL were loci significant in both environments, but with phenotype changes in opposite directions (File S1). The percentage variance explained by each locus and all the loci in the phenotype were calculated using the “fitqtl” function in R/qtl package (Broman *et al.* 2003; Broman and Sen 2009).

### Calculating AP in mapping data

To identify loci with AP, only scale and crossover loci that had GEI mapping p-value < 0.1, slope QTL mapping p-value < 0.05 and *t*-test p-value < 0.005 in each environment were retained. The number of scale and environment specific loci identified in any environment present as a crossover within 60 kb were counted. The degree of antagonism was calculated as the percentage of crossovers among scale or environment specific loci. See File S1.

### Sequence analysis and balancing selection

*S. cerevisiae* and *S. paradoxus* gene sequences from SGRP strains (Liti *et al.* 2009) were downloaded from http://www.moseslab.csb.utoronto.ca/sgrp/blast_original. Sequence alignment, estimating maximum likelihood tree (1,000 permutations) and Tajima’s D were done using MEGA 6.06 (Tamura *et al.* 2013).

### Transcript analysis

Transcript data for the Ras pathway mutants: *ras2(v19)*, *ras2(a22)*, and *ira2* was described in a study by Carter *et al.* (2006). These data were downloaded from the GEO database (accession no. GSE2927). Genes whose transcript levels showed more than a 30% change were selected as those affected by the up or down regulation of the Ras pathway. Genes common across all three mutants of the Ras pathway were analyzed in the SGRP strains (see File S1). Transcript data for SGRP strains (Liti *et al.* 2009) was downloaded from a study by Skelly *et al.* (2013) and hierarchical clustering was carried out using Euclidean distances for expression values. Further, 100 permutations were carried out by randomly selecting the same number of genes to determine whether a similar division of genes occur.

## Results

### Genes involved in cellular organization show maximum trade-offs in deletion analysis

To compare trade-offs across various favorable and nonfavorable conditions, the homozygous nonessential gene deletion collection in yeast, phenotyped for growth in glucose, oxidative, and DNA-damaging environments (Hillenmeyer *et al.* 2008), was analyzed (see File S1). To estimate antagonism, pairwise environment comparisons were made, and a total of 10 environmental pairs were tested (see File S1). Genes whose deletions had better growth (> 0.5 SD) than the population mean in one environment and poor (< 0.5 SD) in the other or vice versa were classified as antagonistic (Table S1, see the section *Materials and Methods*). Similar analysis was carried out using ± 0.75 SD as a cut off (Table S1, see *Materials and Methods*). The percentage of genes showing antagonism ranged from 0.14-6.4% for 0.5 SD and 0.03–3.23% for 0.75 SD (Table S2).

Genes showing trade-off between growth in rich *vs.* a stressed condition (for example, YPD-hydrogen peroxide and YPD-hydroxyurea) were enriched in various mitochondrial functions, including mitochondrial organization, mitochondrial genomic maintenance, mitochondrial translation, *etc*. Very few genes (0.14% with 0.5 SD and 0.03% with 0.75 SD cut-off) showed trade-off between growth in YPD and 4NQO (which is a DNA-damaging agent), and they were enriched in DNA damage repair. Interestingly, genes enriched in various mitochondrial functions showed trade-offs not only between growth in a rich condition (YPD) and various stresses but also between different types of stresses (hydrogen peroxide-hydroxyurea, hydrogen peroxide-4NQO, paraquat-hydroxyurea, 4NQO-hydroxyurea and paraquat-4NQO, see Table S1) for both ±0.5 and ± 0.75 SD cut offs. These results indicated that there exists a broad range of mitochondrial functions that could benefit the phenotype exclusively in certain conditions through mechanisms, which are detrimental in other conditions. An exception to this was comparison between hydrogen peroxide and paraquat, both of which are known to act through separate pathways. Genes showing trade-off between these conditions were enriched in chromatin organization and modification along with regulation of various metabolic processes (Table S1).

### Abundant antagonism in the recombinant population

To compare the abundance and genetic regulation of the trade-offs in the deletion collection with natural populations, QTL mapping was conducted in a large biparental population (derived from a cross between BY and RM strains) grown in diverse environments (Bloom *et al.* 2013). QTL mapping in each environment identified alleles, including small effect QTL similar to the original study (Bloom *et al.* 2013), which explained up to 50% of the overall variation (Table S3).

**Figure 1:**
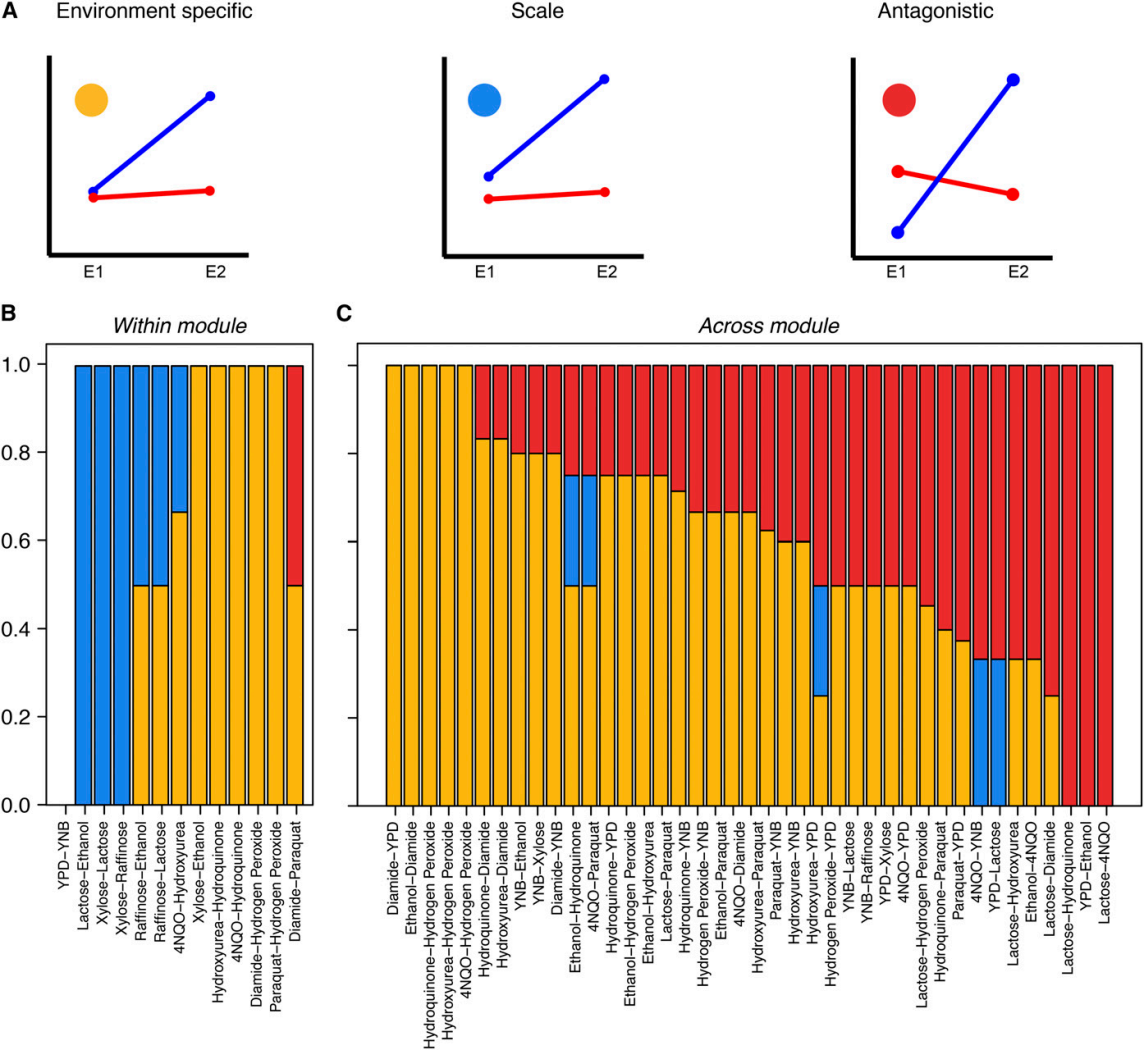
Distribution of the three GEI categories. (A) Schematic reactions norms of QTL for the three GEI categories. Red and blue lines indicate different parental alleles. E1 and E2 (x-axis) are the two environments. The y-axis is the normalized growth phenotype. The colored circles in each graph indicate separate GEI categories. Yellow circle: environment specific QTL, *i.e.*, those that are significant in either environments but not in both; blue: scale QTL, *i.e.*, those that are significant in both environments and for which the allelic order of the mean phenotype is in the same direction in both environments; red circle: antagonistic QTL, *i.e.*, those that are significant in both environments, with opposite allelic order of the mean phenotype, namely that whereas the one (blue) allele is better in one environment, the other allele (red) is better in the other. The same color legend is followed in B, C. (B) “Within module” (similar) environmental pairs, arranged in order of increasing number of antagonistic QTL. (C) “Across module” (dissimilar) environmental pairs, arranged in increasing number of antagonistic QTL.

Mapping GEIs, a highly sensitive way to detect QTL contributing to variation between a pair of environments (Bhatia *et al.* 2014), was used to identify alleles with antagonistic effects. This was done using two methods: R/qtl GEI and Slope QTL mapping for various environmental pairs (See File S1 for the environmental pairs, Table S4, see the section *Materials and Methods*). In R/qtl GEI mapping, the environment is considered a covariate, and a locus is significant if the phenotype of the two alleles varies with the environment. In contrast, in Slope QTL mapping, a new phenotype, namely an estimate of the slope of the mean of the phenotype measure between pairs of environments, was used for each allele. Such a representation is called “reaction norms” (Pigliucci 2005). Mapping was then carried out using “slope” as a novel phenotype, and a locus was considered significant if the slopes for the two alleles had significantly different values. The majority (81.2%) of the slope QTL overlapped with the GEI QTL; and all the GEI QTL were significant in the slope QTL mapping (permutation cut-off p-value < 0.05). These GEI loci were then categorized as: environment specific (a locus with an effect in only one environment), scale (a similar effect but varying in magnitude in both environments), or antagonistic (with opposite effects in the two environments with the RM allele performing better in one and the BY allele performing better in the other environment, Table S5, see the section *Materials and Methods*). When represented as reaction norms, nonparallel nonintersecting slopes depict environment specific or scale interactions, whereas intersecting slopes represent antagonistic interactions (Figure 1A).

**Figure 2:**
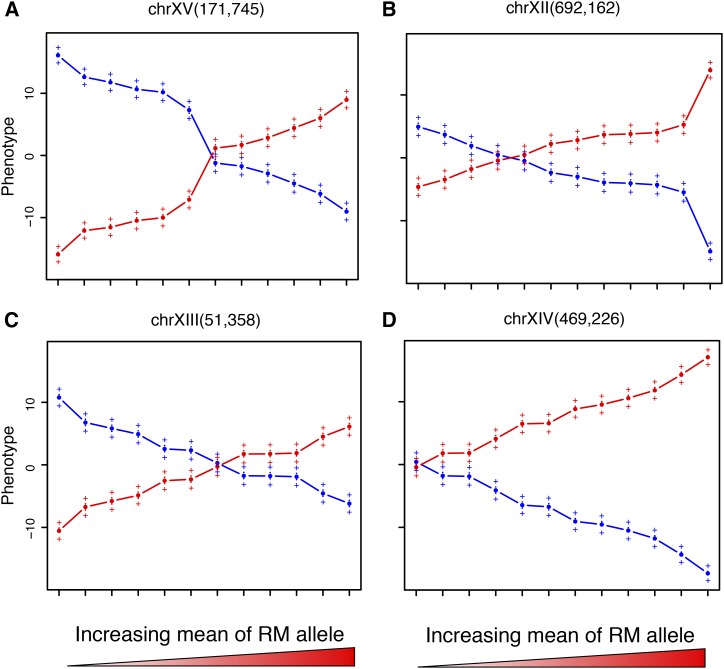
Effect plots of representative loci across 12 environments. Representative loci: (A) chrXV(171,745) [*IRA2*], (B) chrXII(692,162), (C) chrXIII(51,358), and (D) chrXIV(469,226) [*MKT1*]. BY allele effects are indicated as blue, RM allele as red. The environments on x-axis are arranged according to increasing mean of the RM allele. The y-axis is the normalized growth phenotype. The error bars indicate ±1 SE.

Comparison of the antagonism observed showed that the percentage of loci showing a trade-off was the least in the deletion collection (maximum 6.4% using 0.5 SD cut off), followed by 21% (considering all pairs) in independent QTL mapping (Table S2). The greatest proportion of loci were found in GEI mapping (about 39%), which can be attributed to the higher sensitivity of GEI mapping. 51.6% QTL showed antagonism across at least one environment (p-value < 0.05, Figure S2 and Figure S3). Figure 2, A−C shows examples of loci exhibiting AP across various environments. These results suggested an abundance of trade-offs of various polymorphic alleles in natural populations. However, although abundant, the relative magnitude of the trade-offs between allelic variants was similar to that of the deletion strains (Figure S3). Consequently, our results did not support a model, which posits comparatively small effect trade-offs in natural populations. In addition, although both the deletion collection and the recombinant population are synthetic datasets, there is a fundamental difference in the scale of pleiotropy between them. Pleiotropy seems to act at the gene-level for the deletion collection and at a mutation-level for the recombinant population. Because mutation-level pleiotropy is more relevant for evolutionary history (Paaby and Rockman 2014), a greater prevalence of AP in recombinant population compared with the deletion collection indicates that AP plays a role in adaptation.

To test whether this abundant antagonism in recombinants was specific to certain environmental pairs or whether adaptation always results in alleles showing AP, independent of the selection conditions, we divided all pairwise interactions into two groups. Environments considered in the study included rich conditions (YPD, YNB), poorly fermentable/nonfermentable carbon sources, and various oxidizing and DNA-damaging agents. Although each condition is known to invoke a unique response, from previous studies we know that similar stresses, such as two nonfermentable carbon sources, have a similar response, which is different from the response to two oxidizing agents (Broach 2012). We defined “Within module” environments as those that have pairwise similar GEIs, for example, YPD-YNB, xylose-lactose, diamide-hydrogen peroxide, *etc*. We defined “Across module” environments as those that are dissimilar with respect to GEIs, for example, YPD *vs.* different kinds of stress environments, such as nutritional or oxidizing stress, *etc*. Two different kinds of stresses also were included in the “Across module” category, namely lactose−hydrogen peroxide. The amount of antagonism observed was significantly greater in the “Across module” than the “Within module” environment (Figure 1). High antagonism throughout the “Across module” environment suggested that AP results not just from adaptation in favorable *vs.* nonfavorable conditions but also when the organism is adapting to a variety of nonfavorable conditions. Although previous studies have shown how mutations acquired during adaptation in a particular environment could show trade-offs in functionally diverse environments (Kvitek and Sherlock 2013), our results exhibit a high abundance of such trade-offs and how they could be identified with the use of a synthetic recombinant population. In addition, our result suggested that mutations, which show trade-offs, often are retained in parental strains.

Because our analysis did mapping in a diverse spectrum of environments, we were able to study the effect of each putative locus in different conditions. For example, we found that a large effect locus at chrXIV(469,226) containing *MKT1* performed poorly with the BY allele than with the RM allele (Figure 2D) but exhibited very little GEI, *i.e.*, it had a similar effect in almost all environments. In contrast, a chrXV locus containing *IRA2* (Smith and Kruglyak 2008) exhibited a trade-off between nonstress and oxidative and DNA-damaging stress. The BY allele for the *IRA2* locus performed better in all nutrition conditions lacking oxidizing and DNA damaging stresses, whereas the RM allele performed better in all other stresses (Figure 2A).

### Epistatic interactions play a major role in regulating trade-offs

Epistasis, which has been proposed to play a significant role in GEIs, has to date been largely undetected, most likely due to lack of power (*i.e.*, due to small sample sizes) required to resolve genotype × genotype × environment interactions. In our study, a large sample size and the use of the slope as a phenotype enabled the detection of such epistatic effects (see the section *Materials and Methods*). Twenty-five environment-dependent, two-QTL interactions were identified (Table S6). A majority of these interactions (20/25) were not significant within either environment but showed an effect on the slope when different environmental pairs were compared. Similar to single QTL mapping where GEI identified QTL that were not significant in either environments, this finding suggests that, genetic interactions, which are insignificant in a single environment, might play a role in adaptation across environments. In other words, small, undetectable effects in each environment might play a significant role in the variation of phenotypes in a different environment.

**Figure 3:**
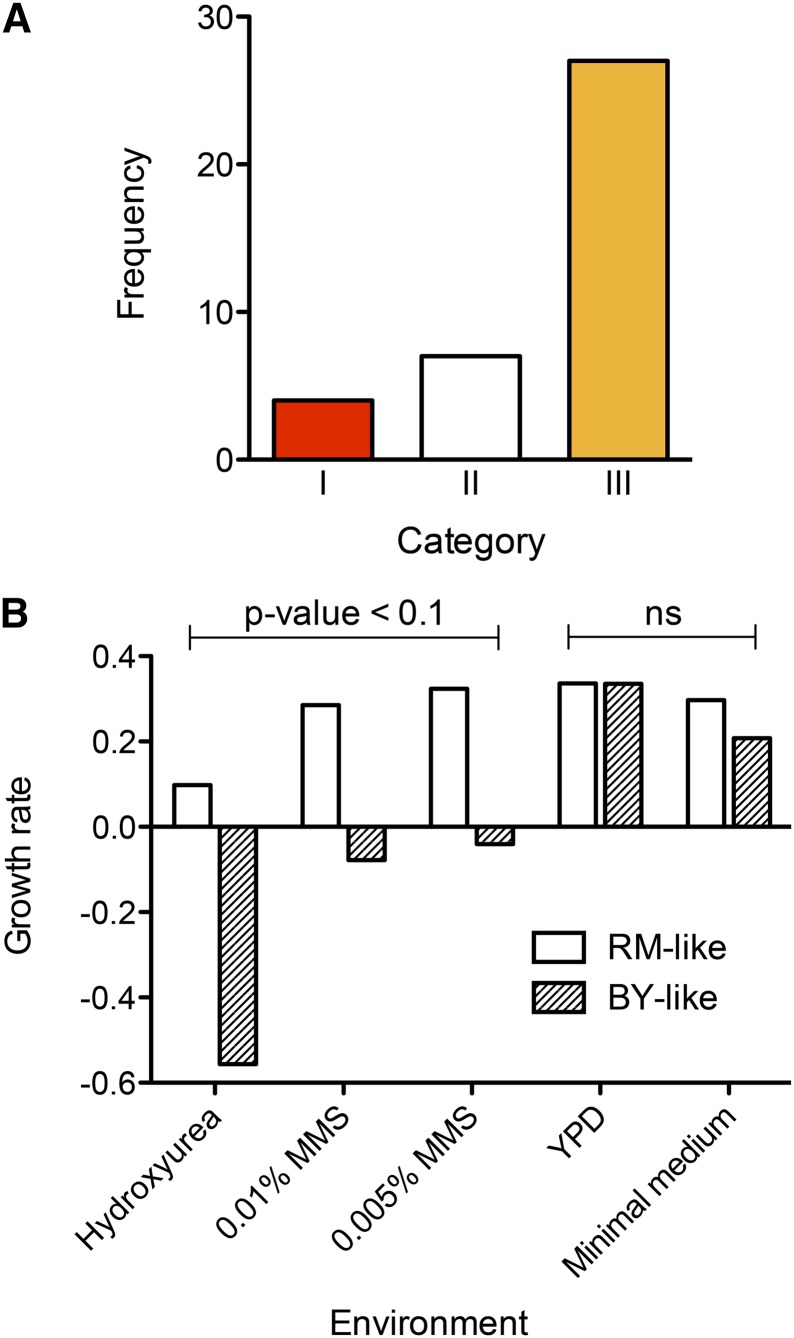
Lack of trade-offs in natural populations. (A) Antagonistic pleiotropy in SGRP strains. The mean growth of strains in the SGRP population was calculated in each of the four environments. A strain with a value beyond ± 0.5 SD range was considered significantly different and strains were categorized as having a better/poor state for each of the four environments. Category I shows the number of strains showing trade-offs across at least one environment, *i.e.*, greater than +0.5 SD and lower than −0.5 SD in at least one environment each; category II represents the number of strains within ± 0.5 SD of the population mean for all environments; and category III are the strains varying more than 0.5 SD in at least one environment without showing trade-offs. (B) Normalized growth phenotype of SGRP *S. cerevisiae* strains grouped into BY- or RM-like classes on the basis of homology of *IRA2* in different environments (x-axis). The environments are YPD, hydroxyurea, methyl methane sulfonate (MMS), and Minimal Medium. The y-axis is the normalized growth rate (Warringer *et al.* 2011).

The greater antagonism of allelic variants identified in our study (see previous section) implied that there may be a negative association between growth and stress resistance in natural strains. To study this further, we compared the growth for 38 diverse SGRP strains (Liti *et al.* 2009) in rich growth and DNA-damaging conditions (Warringer *et al.* 2011). Only four strains showed antagonistic growth across at least one environmental condition (Figure 3A, Table S7, see the section *Materials and Methods*). This lack of AP in natural populations suggested compensation or resolution of trade-offs over the course of evolution in natural isolates. Because a large number of alleles showed antagonism in the recombinant population, the lack of trade-offs observed in natural isolates suggests that different evolutionary trajectories were taken by the strains while adapting to the same stresses, which resulted in the accumulation of mutations with antagonistic effects detectable in crosses, but without a net difference in adaptation for the natural isolates.

**Figure 4:**
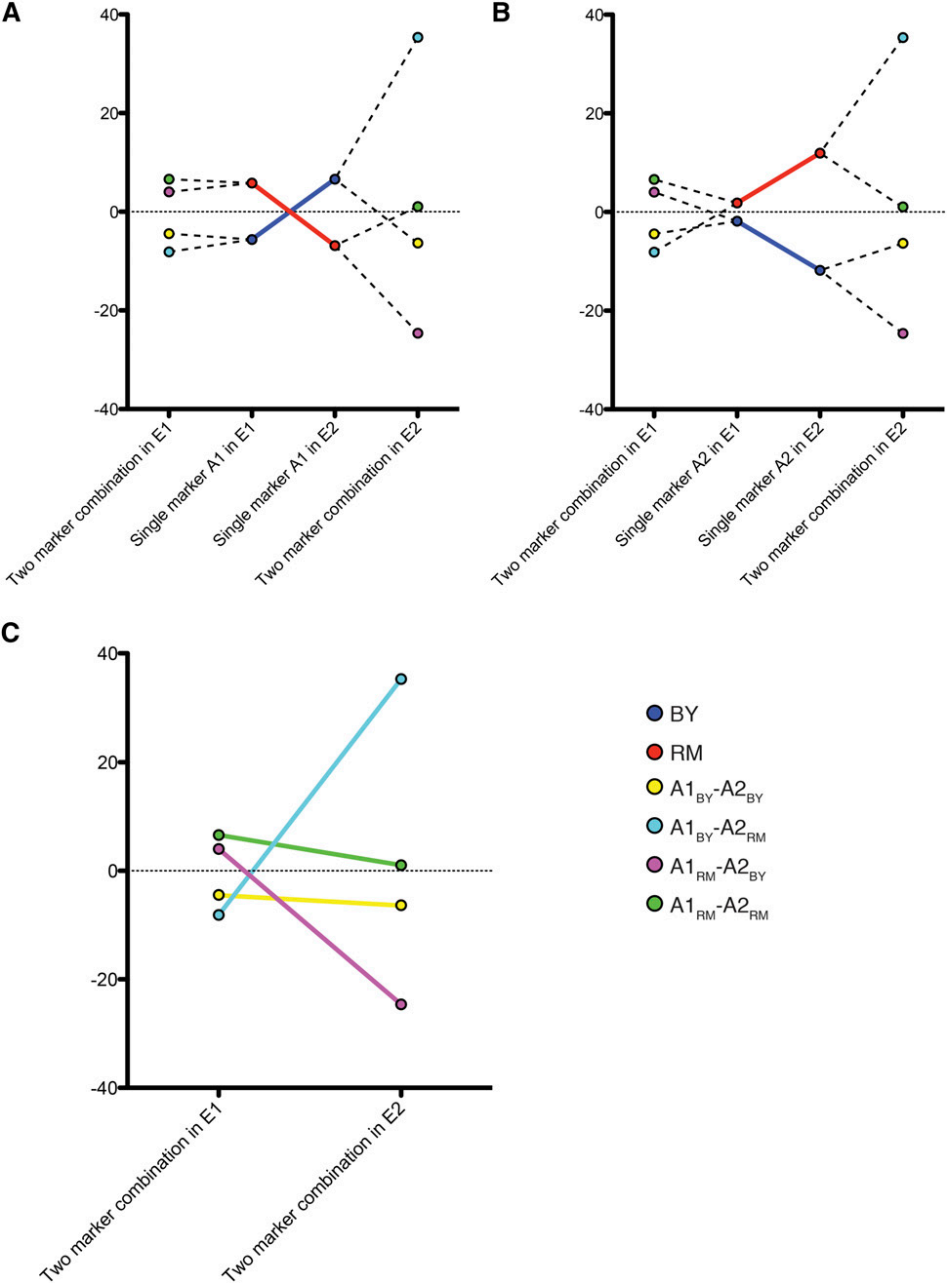
Example of an environment dependent two-QTL interaction. (A, B) Respective reaction norms of the two markers A1 and A2 in the two environments E1 and E2. The x-axis indicates environmental conditions. The y-axis is the normalized growth phenotype. In this example, E1 is hydroxyurea, E2 is YPD. Marker A1 is chrXIV(377,033) and A2 is chrXIV(470,303). Solid lines indicate reactions norms of the two markers in E1 and E2 (A, B respectively). Dashed lines demonstrate division of population carrying one allele into two groups based on the alleles of the other marker. (C) Reaction norms of the four two-marker combinations. YPD, yeast extract peptone dextrose.

To achieve optimal fitness, a mutation with a conditionally detrimental effect in a population can either be selected out, or else other mutations may accumulate (mutation accumulation) to compensate the antagonism (Orr 2000; Pavlicev and Wagner 2012; D’Souza and Wagner 2014). Although difficult to study and predict in natural strains, such mitigation of trade-offs through various genetic interactions can be explored in a recombinant population where these compensatory interactions become uncoupled. We analyzed environment dependent two-QTL interactions to detect such resolutions. The means of segregants containing various marker combinations were compared, and the difference between the means was used to estimate trade-offs. We found that the occurrence of trade-offs was lower for homozygous parental combinations of the two markers *i.e.*, BY-BY and RM-RM (Table S6). In heterozygous crosses, in 18 of 25 instances, either one or both markers involved in interactions showed a crossover. However, in homozygous parental combinations, the parental combinations of the two markers showed antagonism in only 9 of 25 instances, supporting resolution of trade-offs (Fisher’s exact test < 0.01, Figure 4). Furthermore, in 23 of 25 two-QTL interactions, at least one allelic combination pair showed an antagonistic interaction (Table S6). This increased trade-offs in recombinant segregants provided further evidence for the resolution of antagonistic effects during evolution.

### Signature of contribution of Ras/PKA pathway in driving adaptation

Alleles showing a trade-off could be maintained in natural populations as the result of balancing selection, *i.e.*, depending on the environmental condition the strain adapted in, one or the other allele of the same gene can be under positive selection (Hedrick 1999). Situations that give rise to such balancing selection may be opposing effects of alleles in distinct stages in the life cycle or else environmental conditions that appeared periodically during its evolutionary history. The genes identified in BY × RM segregants in various mapping studies (Fay 2013) were evaluated for signatures of balancing selection by calculating Tajima’s D (Tajima 1989) in the SGRP strains, consisting of *S. cerevisiae* and its sibling species *S. paradoxus* (Liti *et al.* 2009). *IRA2* was the only gene that showed positive balancing selection for both species as well as trade-offs in GEI mapping. *IRA2* is a negative regulator of the Ras pathway, which is a major growth signaling pathway in yeast and functionally conserved across taxa (Ballester *et al.* 1990; Martin *et al.* 1990; Xu *et al.* 1990; Broach 2012). Although the polymorphic nature of *IRA2* has been discussed previously (Smith and Kruglyak 2008), our analysis suggests that the BY and RM alleles of *IRA2* represent two dominant allele frequencies in diverse yeast strains (File S1). Both BY and RM *IRA2* allele occupied central positions in the two clusters respectively. 

**Figure 5:**
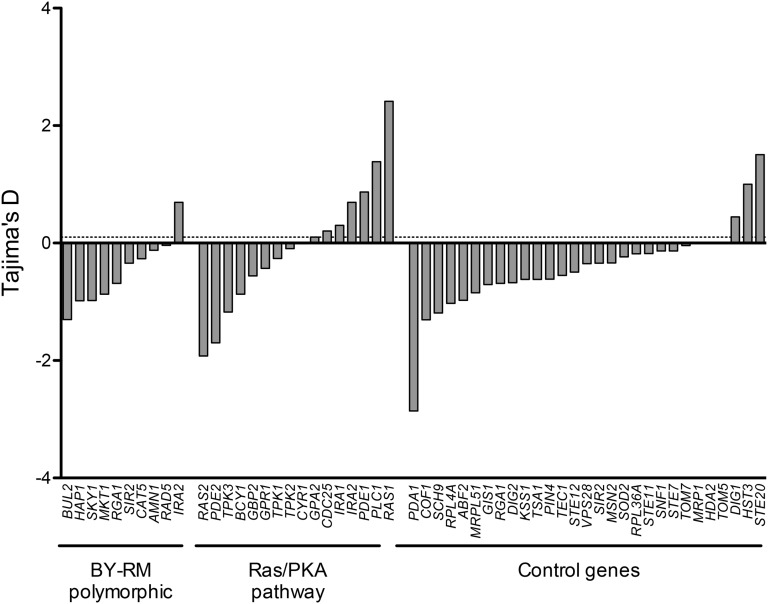
Tajima’s test of neutrality for different categories of genes in *S. cerevisiae*. The first group of genes is polymorphic between BY and RM, the second group is the regulatory genes of the Ras/PKA pathway, and the third group is the control set of genes including mitogen-activate protein kinase pathway, transcription factors and other genes showing a trade-off in deletion collection. The dashed line indicates a cut-off of 0.1. PKA, protein kinase A.

To determine whether this trade-off associated variation is specific to *IRA2* or is a general property of the Ras/PKA pathway, we repeated the Tajima’s D analysis for the major Ras pathway genes. The D values for the Ras pathway genes were compared with those of a control set consisting of stress responsive transcription factors and genes which were identified in the deletion collection AP analysis (see the subsection *Genes involved in cellular organization show maximum trade-offs in deletion analysis*) as well as an alternate signaling MAP kinase pathway (Figure 5 and Table S8) in both *S. cerevisiae* and *S. paradoxus*. This diverse control set included genes both specific to yeast as well as those conserved across taxa. We found that a high percentage of the Ras/PKA pathway genes (5 of 16, Fisher’s exact test, p-value < 0.1) showed balancing selection in both species compared with the control set. Furthermore, the Ras pathway genes were significantly enriched (Table S9) in the list of genes identified in various mapping studies in yeast (Fay 2013). This evidence together indicated that multiple functional variants of the Ras/PKA pathway are present in natural yeast populations, which either independently or in combination contributed to their adaptation in diverse conditions.

To test whether polymorphic Ras/PKA pathway genes indeed result in altered Ras pathway signaling in the natural isolates, we compared transcript profiles of Ras/PKA targets in the SGRP strains. Altering Ras/PKA genes has a large effect on the transcriptional state of its downstream genes (Jones *et al.* 2003; Carter *et al.* 2006; Zaman *et al.* 2008; Broach 2012). The targets of Ras were identified by comparing transcriptional changes induced by mutations in *RAS2* and *IRA2* (see the section *Materials and Methods*). Approximately 300 selected genes were then clustered on the basis of their expression in specific natural isolates (Skelly *et al.* 2013). We found that the transcripts clustered in two distinct groups, consisting of 113 and 176 genes each (p-value = 0.1, Figure S4), and indicating differential Ras/PKA signaling in the natural strains. Although the complete set of 300 genes was not enriched for any GO category, of the two clusters, one with 113 genes was enriched for chromatin organization and the other containing 176 genes was enriched for oxidation-reduction processes, indicating a difference in the functional basis of the two clusters (Table S9). To test whether this altered Ras signaling translates into a trade-off between growth in rich and stressed conditions, 36 SGRP *S. cerevisiae* strains were classified either as BY- or RM-like, based on their *IRA2* sequence. Their growth was compared in glucose and DNA damaging agents (see File S1). While there was no significant difference in growth between the two groups in glucose, the RM-like group performed significantly better in both DNA-damaging conditions (Figure 3B, *t*-test p-value < 0.1). Analysis of other polymorphic genes (such as *MKT1*, *RAD5*, and *PHO84*) did not cluster the two alleles separately (File S1). Although 36 strains is a small number to identify association of a polymorphism with phenotype (Nieduszynski and Liti 2011), our results indicated that the two groups of strains differed significantly in stress inducing media, whereas they did not show a corresponding effect on growth in glucose rich conditions, which is the prototypic signature of balancing selection.

## Discussion

Although AP has been identified across multiple traits, its evolutionary relevance remains elusive. Using a meta-analytical approach, we studied AP in three diverse experimental systems, which was possible because of the genetic accessibility of yeast as a model system. We showed that, despite the presence of a large number of alleles showing antagonistic effects in the segregating population, there was little evidence of trade-offs in natural isolates. This is probably because strains had evolved compensatory genetic changes over time to reduce the fitness cost associated with AP. Although adaptation to an environment can be brought about by varying multiple regulatory and metabolic processes, signaling pathways can provide greater phenotypic flexibility, allowing both plasticity of response in a new environment and resolution of the associated loss in fitness by cost-sharing it among many genes. Our study suggested that although a variety of cellular processes could regulate fitness traits under selection, passing AP fitness costs to downstream genes, and thereby retaining variation in signaling pathways (such as the Ras/PKA pathway) seems to be a preferred approach used by adapting populations over the course of evolution (Kvitek and Sherlock 2013; Lorenz and Cohen 2014).

Although both the deletion collection and the segregant populations showed AP, it was more prevalent in the latter, implying that genes showing trade-off tended to be polymorphic in natural populations. However, the poor overlap between genes showing trade-offs and functional allelic variants in natural populations (9 of 93, Table S9), and the presence of purifying selection on deletion collection genes which showed trade-off (Qian *et al.* 2012) indicated that trade-offs and adaptation in natural populations is regulated by genes distinct from those identified by deletion analysis (Nogami *et al.* 2007; Fraser *et al.* 2012). This observation suggested that conclusions about the evolutionary role of genetic variants identified by gene deletion studies should be treated with caution.

As shown by various laboratory-based evolution studies (Kvitek and Sherlock 2013), a high prevalence of AP in the segregant population suggested that as strains evolve, they accumulate beneficial mutations to adapt to the immediate environment, independent of the antagonistic effects in other environments. However, how populations showing AP escape its effects in detrimental environments is not known (Kvitek and Sherlock 2013). By comparing QTL and two-QTL mapping, we propose that a common mechanism to reduce this cost could be to accumulate new mutations to mitigate the effect of AP and allow the organism to adapt to the environment where it was previously deleterious. This mechanism explains why, despite a lack of overall trade-offs between divergent strains, antagonistic alleles from both parents can be identified in a recombinant population. The reason for this is that in the natural strains, trade-off was mitigated over time by passing on fitness costs to many loci, which become visible when they become unlinked in the cross. This unlinking of resolved trade-offs can also explain the high transgressive variation observed in such segregating populations (Tanksley 1993). In a novel environment, a meiotic event between two such strains would disperse multiple adaptive alleles (Charlesworth 2012). Hence, this process may also be a mechanism adopted by populations to maintain genetic memory of trade-offs, allowing the organism to remain poised to adapt to unfavorable conditions without a detrimental effect. Conversely, if antagonistic epistasis mitigates large effect trade-offs, then a hybrid would have a lower overall fitness than either parent (Dobzhansky 1970). Such heterozygote inferiority could then encourage reproductive isolation of the two strains, ultimately leading to strain or species diversification (Dettman *et al.* 2007).

The Ras/PKA pathway regulates multiple phenotypes across taxa, including reproduction, development, longevity and metabolism. Activation of the Ras pathway results in active growth, and consequently, it is down-regulated when proliferation is not preferred or required, such as when cells are under stress or are differentiated. However, although the Ras pathway antagonistically regulates cellular growth and robustness, it provides scope for compensation of defects associated with these phenotypes. We show that some natural yeast isolates employ the Ras pathway during adaptation to achieve higher stress resistance without the associated growth defect. In humans, ~20% of all tumors have Ras mutations (Downward 2003). Although overproliferation would make a (malignant) cell vulnerable to stresses resulting in programmed cell death and eventual tumor regression, mutations in this pathway (EGFR pathway) can bypass this by developing survival adaptation (Rajput *et al.* 2007). Similarly, although mutations in various signaling pathways (Ras, TOR, and their downstream transcription factors) increase longevity at the cost of reduced growth, yeast lacking *RAS2* and worms with *daf-2* mutation overcome this growth defect (Longo and Finch 2003; Longo 2004). This consistent ability to balance various antagonistic phenotypes could explain the “conserved yet polymorphic” nature of such a central pathway, not just in yeast but also across taxa (Alvarez-Ponce *et al.* 2011; Jovelin and Phillips 2011; Luisi *et al.* 2012). This ability to fine-tune various fitness traits, most likely through different epistatic interactions, may be the result of the high connectivity of this pathway (Lee *et al.* 2008; Olson-Manning *et al.* 2012). Similar effects also are seen on other pathways that are hubs in the cellular machinery, such as the p53 pathway (Atwal *et al.* 2007; Hu *et al.* 2007; Ungewitter and Scrable 2009). Indeed, as organisms became complex, the requirement to balance multiple antagonistic phenotypes might be the reason that gene and protein networks developed regulatory hubs controlling basic cellular pathways, such as cell cycle, apoptosis, autophagy and anoikis. One may also speculate that partially resolved AP in such key pathways may be the reason for rapid speciation following an extinction event, as organisms scramble to adapt to novel niches and environmental conditions which open up as recombinants suddenly find themselves adapted to the new situation.

Our results show how the effect of a QTL on regulating a phenotype is heavily dependent on both the environment considered (pleiotropy and trade-off) and the genetic background (accumulated mutations). This results in a high environment and strain dependence of effect of QTL observed both in yeast and other model systems like *Drosophila* resulting in a lack of reproducibility of QTL identified across experiments (Macdonald and Long 2004; >Cubillos *et al.* 2011). Abundant environmental AP and its resolution in different natural isolates provide an explanation for identification of different QTL across various studies. Although difficult in other model systems, accessibility to gene level resolution and genetic manipulation techniques like reciprocal hemizygosity analysis, RHA (Steinmetz *et al.* 2002), in yeast can help predict and test the reproducibility of QTL regulating various phenotypes. For example: *MKT1* is a regulator of stress response with pleiotropic effects on growth, and is non-functional in the laboratory strain BY. As a result, it is identified in all synthetic crosses which have BY as one of the two parents (Steinmetz *et al.* 2002), which includes a recombinant population derived from a cross between BY and a clinical isolate YJM789. Other such examples include *MAL13* and *FLO8*, both of which are nonfunctional in the laboratory strain, probably resulting from decades (many million generations) of domestication on a rich medium, which make them identifiable in various recombinant populations. In contrast, *RAD5* is a DNA helicase involved in DNA damage tolerance and is nonfunctional in RM making it more susceptible to DNA damaging stress. It is therefore, not identified in a cross between BY and a clinical isolate (YJM789) or an oak strain (SK1) (Gerke *et al.* 2006). Although our analysis of a cross between BY and RM has identified *IRA2*, and hence the Ras/PKA pathway, as a key player in the regulation of growth related AP, analyzing recombinant populations derived from other diverse parental strains, and using a larger natural population may identify other pathways contributing to AP regulations and provide a deeper understanding of the role of trade-offs in determining adaptation and evolution.

In conclusion, our study exemplifies the power of using the diverse sets of experimental systems available to study yeast to address a fundamental question, which is to understand the mechanisms underlying maintenance or resolution of AP. In our approach, the use of three different experimental systems was key to our ability to interpret and elucidate possible mechanisms responsible for the observed inconsistency in AP abundance seen in different studies. Our results suggest that over the course of evolution, mutations accumulate to mitigate trade-offs, preventing the detection of AP in natural populations. However, such resolutions can be identified using methods presented here by studying recombinant populations, where these resolutions become visible due to uncoupling of genetic interactions. Our findings suggest that studying various complex disorders using an evolutionary and adaptive perspective may be a fruitful way to understand mechanisms underlying their regulation and origins.
